# A Clinician’s Guide To Effectively Transitioning and Transferring Care For Pediatric Patients With Inflammatory Bowel Diseases From The Pediatric To Adult Gastroenterologist

**DOI:** 10.1007/s11894-024-00936-6

**Published:** 2024-07-16

**Authors:** Jessica N. Barry, Jonathan D. Moses, Sandra C. Kim

**Affiliations:** 1 Cleveland Clinic, Division of Pediatric Gastroenterology, Hepatology and Nutrition, Cleveland, OH USA; 2 Stanford Medicine Children’s Health Division of Gastroenterology, Hepatology, and Nutrition, Palo Alto, CA USA

**Keywords:** Inflammatory bowel diseases (IBD), Crohn’s disease, ulcerative colitis, transitioning of care, young adults

## Abstract

**Abstract:**

**Purpose of Review:**

Transition of care for pediatric patients with inflammatory bowel diseases (IBD) is a continuous, dynamic process that takes place over several years with a coordinated approach executed by a multidisciplinary team. We review the concepts, tools, and research in effective transitioning and transfer of care for adolescent/young adult patients with IBD.

**Recent Findings:**

Given the constraints within the healthcare system, effective transitioning can be challenging to implement in everyday clinical practice. Different barriers include resources and expertise in effective transitioning by pediatric and adult gastroenterology healthcare providers and the impact of non-gastrointestinal issues facing young adult patients who are learning to manage and coordinate all aspects of their medical care and health maintenance. Factors that facilitate successful care transitioning and transfer include structured transitioning programs, utilization of validated transition checklists, and IBD medical summaries.

**Summary:**

Proactive transitioning by pediatric gastroenterologists in partnership with their emerging young adult patients with IBD leads to better clinical and psychosocial outcomes and ultimately, effective transfer of care to adult gastroenterology. By utilizing utilize comprehensive transition assessment tools and medical summaries in partnership with their patients, pediatric and adult gastroenterology teams can better prepare patients as they transfer to independent care and health maintenance.

## Introduction

Transition of care from pediatric to adult gastroenterology is the key process for eventual and successful transfer of patient care from one provider to another. It is a dynamic process defined as a planned movement of adolescents and young adults with chronic physical and medical care needs from child-centered to adult-oriented health care systems with a purposeful approach [1••]. Successful transition requires education, communication, and strengthening of self-management skills with the ultimate goal of self-efficacy and independence for the emerging young adult living with chronic illnesses like IBD. The North American Society for Pediatric Gastroenterology, Hepatology and Nutrition (NASPGHAN), the European Crohn’s and Colitis Organization (ECCO), and the Crohn’s and Colitis Foundation have issued specific statements regarding the transition of care for adolescents with IBD. NASPGHAN recommendations for the practitioner include the following: [1••] The pediatric gastroenterologist should begin seeing adolescent IBD patients without their parents around 16 years of age to build a relationship promoting independence; [2•] Introduce the patient and family to the concept and benefits of transition; [3] Identify a skilled gastroenterologist who cares for young adults and recognizes the different set of expectations that young adults with childhood-onset IBD have versus those recently diagnosed with IBD; [4] Prepare a detailed medical letter and brief medical summary for the new adult gastroenterologist; [5] Recognize the timing of transition and transfer requires flexibility due to individual needs and circumstances. These guidelines address several issues adolescents and young adults (AYA) with IBD encounter, including the process of transfer from parental oversight to self-reliance and adapting to the change in care environment of a nurturing medical care model commonly seen in primary pediatric care practices.

The transition process requires purposeful involvement by parents/guardians and pediatric health-care provider teams (including physicians, nurses, and additional health care staff) with active engagement of the AYA patient to facilitate successful transfer of care to an adult subspecialist [2, 3]. In the care of patients with inflammatory bowel diseases (IBD) progressing from pediatric to adult gastroenterology care, it is essential for healthcare providers to be knowledgeable and proficient in the concepts of transition and transfer as outlined in Table 1.

**Table 1:** Terminology Transition and Transfer

Term	Definition	Assessment Examples
Transition	Comprehensive process of both acquisition of skills and process of disease self -management and transfer to adult providers.	
Transition Readiness	Acquisition of sufficient skills to transition successfully to adult providers. Possessing the knowledge set, the skills required for self-efficacy, and the social support to successfully transfer to adult medicine and transition to health care independence.	1) Proper medical management 2) Management of emotions 3) Living a meaningful life with IBD
Self-Efficacy	An individual’s perception of their ability to plan and effect actions necessary to manage certain situations.	Self-efficacy at it applies to IBD: 1. Disease self-management (Scheduling appointments, monitoring symptoms, communicating with health care providers and taking medicines)
Health Care Independence	The ability to achieve, make decisions and initiate actions by oneself in regard to one’s healthcare.	
Adherence	The extent to which a person’s behavior including taking medication, dietary choices and lifestyles intersects with recommendations from a health care provider.	

The absence of a “gold standard” or defined best practice of transition creates a challenge to process standardization, measurement of outcomes, and monitoring of sustainment.

If a standard process can be achieved, the outcome expected is facilitation of successful transfer of care from pediatric to adult centered gastroenterology care.

**Fig. 1:**
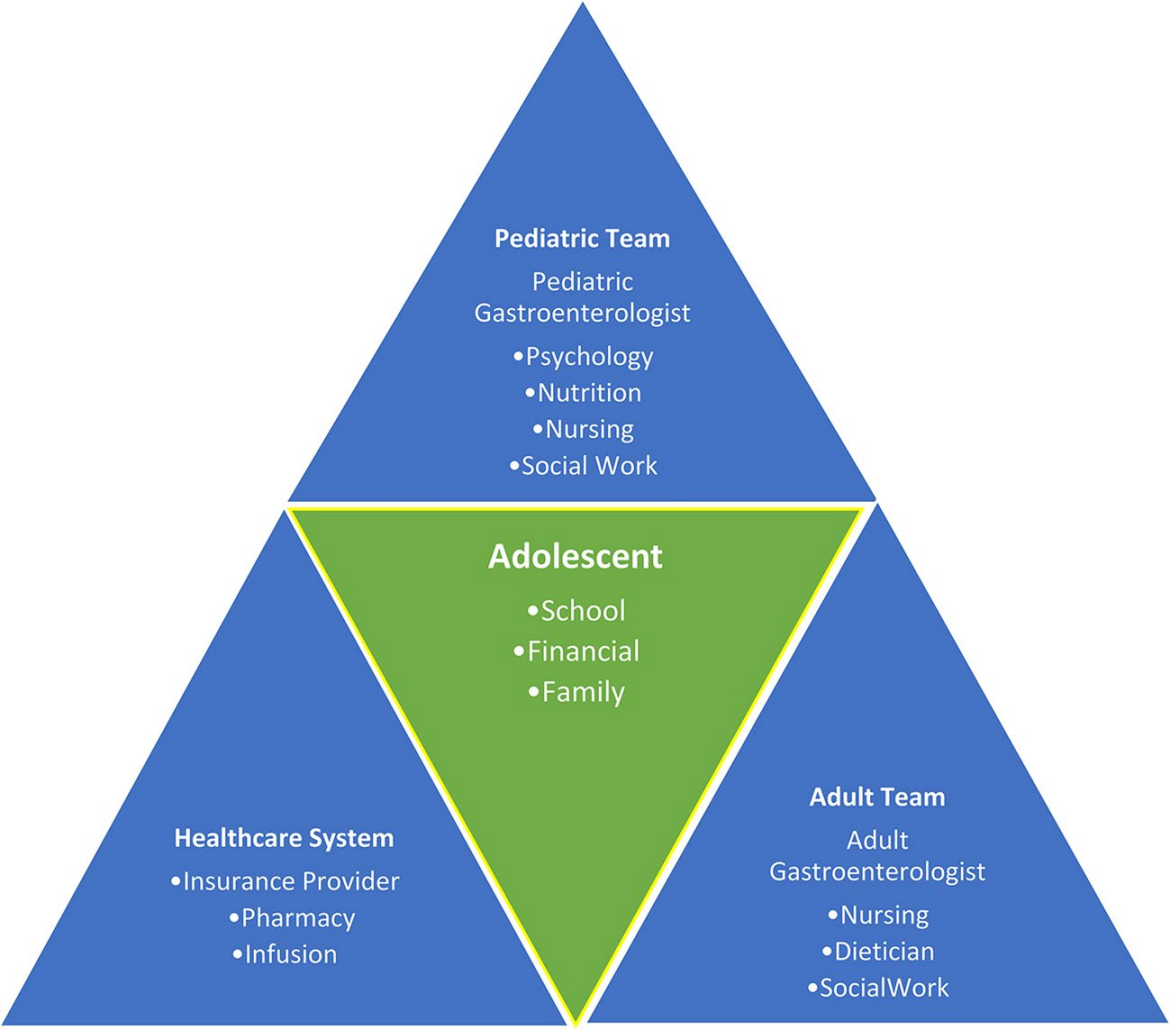
Transition of care stakeholders

An optimal transition care model should be patient - centric, with all stakeholders’ roles clearly defined and ideally linked in a cohesive planning outline to achieve a comprehensive model of care (Fig 1). One approach involves the medical IBD home concept which allows for optimal communication between caregivers and centralized patient care [4, 5]. It is important to consider safety nets and plans of care to monitor and intervene when needed for patients experiencing evidence of unsuccessful transfer. This early intervention and monitoring process could mean the difference between disease exacerbation and negative impacts of a physical, mental, or social aspect for our patients. Hickam, et al. “Outcomes of a Structured Ambulatory Care Health Care Transition Approach in a Large Children's Hospital” is an excellent example of one such successful transition model [6].

Ideally, a transitioning clinical program which focuses on the needs of the young adult patient should be multidisciplinary in nature, incorporating members from pediatric and adult GI care teams. Furthermore, there should be other members of the healthcare team involved including psychology, social work, nurse care coordinators, and dietitians. The TRANSIT study from the United Kingdom showed young adult patients who underwent “structured transition” care (> 2 clinic visits with pediatric and adult GI clinicians) prior to completion of transfer had significantly less disease exacerbations and higher corticosteroid – free remission rates (71% versus 41%) when compared to individuals who had “routine” care (transfer from pediatric to adult GI care directly) [7]. However, only few hospital/academic centers have these comprehensive programs. Therefore, there are ways this can be facilitated. The primary pediatric GI team should begin the process of discussing potential adult IBD – focused GI clinicians before the patients turns 18 years/becomes a legal adult. These conversations can be placed in the context of future plans upon graduation from high school and incorporated as part of future medical, health maintenance, and professional planning (future colleges location; work/vocational goals) and should be planned well in advance of actual care transfer. The eventual formal transfer will most likely occur at various times based on the factors outlined above (pre- or post-graduate education; establishment of a stable occupation/housing).

During the latter part of the care transitioning process prior to formal transfer of care, the pediatric healthcare team should be actively educating and assessing self-care management, and disease knowledge for their young adult patients. Every patient has their unique needs, and each healthcare system and providers have their own unique resources (or relative lack thereof). However, there are a few key items that should be implemented such as a tracking/monitoring system to follow patient progress and ongoing educational materials/resources available to the young adult patient.

## Practical Implementation of Transition

Successful transition of care in pediatric patients with IBD not only requires multidisciplinary support from the medical team (therapies, testing, procedures), but engagement with the patient at an age-appropriate level. It is important to address the psychosocial aspects that impact disease knowledge, health literacy, and self-advocacy. Studies have shown even with the best perceived efforts of pediatric clinicians, there are clear discrepancies between what the pediatric (versus the adult) GI clinician sees with their patients’ readiness to effectively transfer. Furthermore, clinician perception versus defined-measures of health literacy is not well correlated, especially within the realm of patient – reported self – efficacy in disease management. Self-efficacy in disease management is a key component of how a young adult will do managing their disease as an independent adult within the adult healthcare system [8–10]. In clinical practice, there are a variety of tools that can be used to assess differing aspects of transition readiness in adolescent and young adult patients with IBD. These tools are used to facilitate the advancement of disease state and healthcare knowledge of children and young adults with IBD and include the following areas: a) age-appropriate checklists, b) self-efficacy skills, c) disease knowledge, and d) transition readiness [11, 12].

Other factors that need to be incorporated in these discussions and decisions include insurance coverage since this will influence who the young adult will be able to see moving forward. One of the greatest stressors for patients and families alike is the issues revolving around financial issues, especially from insurance and pharmacy benefits manager (PBM) coverage/type, which can impact not only where the patients can obtain clinical care but also infusion and procedure centers, pharmacies, and laboratory facilities. Understanding the impact of these financial factors will decrease the chance there will be lapses in care which has been shown in young adults with chronic illnesses including diabetes and spina bifida [13, 14].

Impact of gender on transition readiness must also be considered. Transitional care of young girls and women with IBD have additional considerations which make the importance of timely transfer of care essential, such as consideration of reproductive health needs. Abrupt care transfer in a patient who is inadequately prepared may be triggered by life events such as pregnancy, or sudden health care needs requiring adult gastroenterology care and monitoring. Amongst American women and girls aged 15-44 years approximately 45% of pregnancies are unintended based on a review of pregnancy rates from 2008-2011, making early planning and patient education essential [15].Additionally, the importance of patient education by care team providers for health maintenance in IBD needs to be actively reviewed and coordinated in multiple areas including, but not limited to, gynecologic screening and reproductive health care needs.

Another critical component of effective care transfer revolves around the final step of formal transfer. Ideally, if the transitioning process preparing the adolescent/young adult patient has been an ongoing, dynamic process throughout the adolescent years, the move towards care transfer should be a natural process rather than one that feels rushed. By age 17-18 years, the healthcare team should prepare the patient and family that the patient will be the one responsible for medical decision making as a legal adult. Whether the young adult patient formally transfers to adult gastroenterology care at this time, or remains with their pediatric gastroenterology team through the initial young adult years (especially given that this also is a time of many other transitions in life (i.e. graduation from high school; matriculation in college/university; start of new job; moving out of the home), the young adult must be able to understand and manage the processes related to effective care. Furthermore, the pediatric healthcare team needs to identify potential barriers to this process including medical stability (are their IBD and other related medical issues under adequate control?), psychosocial readiness, and family concerns.

A key concept for successful care transfer focuses on effective communications both written and direct/spoken, between the pediatric and adult gastroenterology healthcare teams. The ideal transfer of care involves the “warm handoff” with direct communications between the two teams. A comprehensive medical summary which is clearly documented in the medical record is an essential part of this process to ensure that all members of the pediatric and adult healthcare teams have a good understanding of the patient’s medical history. An ideal medical summary should include the following: [16]

1. Date of original IBD diagnosis
2. IBD phenotype
3. IBD – related surgeries, hospitalization, and complications
4. Medication and therapy history including prior adverse reactions
5. Recent labs including therapeutic drug monitoring levels
6. Diagnostic testing results including endoscopies and radiographic imaging
7. Health maintenance items including vaccination records and screening status (tuberculosis; vaccine titers – hepatitis B)

Hait and colleagues have similar recommendations as outlined below to include in the medical summary letter (Table 2) [17].

**Table 2:** Medical summary letter

Disease information: Date of diagnosis, type of IBD; location; severity	
Findings: Laboratory testing; endoscopic evaluations and histology; radiology	
Medical therapies: Dose and duration; prior adverse reactions; reasons for discontinuation	
Surgical history (related to IBD)	
Psychosocial, developmental, and educational issues	

Adapted from Hait et al. [17]

## Age-appropriate Checklists

It is important to allow the young adult patient to be an active participant in these processes (i.e., providing checklists of the necessary skills and educational materials to address the areas of need identified by the assessment tools), improving disease knowledge, and allowing for more active listening by the clinicians. To allow the patients to develop in an age-appropriate manner, various checklists have been developed for use in clinical practice [11, 12, 18]. Each developmental stage typically involves an age range and varies based on each published paper. In the article by DeSilva et al, they advocate for delineating into 11-13 years of age, 14-16 years of age, 17-19 years of age, and 20-23 years of age. The checklist published by Barry et al and the Healthcare Provider Transition Checklist published by the North American Society for Pediatric Gastroenterology, Hepatology and Nutrition (NASPGHAN) early-adolescence (12-14 years of age), mid-adolescence (14-17 years of age), and late adolescence or young adult (over 18 years of age). Tools to approach planning and readiness checklists can help facilitate successful transition. NASPGHAN, the National Alliance to Advance Adolescent Health, transition tool is an example of a transition resource. Assessment aids are available in review of transition readiness for providers and patients (Table 3). (GotTransition.org). Typical questions include the following: [1••] Describe your disease and other associated medical issues related to your IBD; [2•] What are symptoms or other quality of life factors that are associated with your IBD?; [3] What things worsen your IBD and quality of life?; [4] Do you know how to contact your doctor/nurse/healthcare team?; [5] What things are you doing to be an active part of your care?; [6] How do you keep track of your medications, tests, and results?; [15] How much do you know about insurance?; [7] What things are most important for you in this whole process? By addressing these things in a proactive manner, one can increase the young adult patient’s understanding of their disease and self-efficacy and encourage effective shared decision making.

**Table 3:** Summary of transition resources and tools

***Educational resources and transition guidelines for providers***	
**•** “A case-based monograph focusing on IBD: Improving health supervision in pediatric and young adult patients with IBD” (NASPGHAN)	
**•** “Educate, communicate, anticipate: Practical recommendations for transitioning adolescents with IBD to adult health care” [19•]	
**•** Transition of the patient with inflammatory bowel disease from pediatric to adult care: Recommendations of the North American Society for Pediatric Gastroenterology, Hepatology and Nutrition” [2•]	
**•** “Transitioning the adolescent inflammatory bowel disease patient: Guidelines for the adult and pediatric gastroenterologist” [3]	
***Transition readiness assessment and tools***	
*For patients*	
**•** Preparing to transition from a pediatric to adult care practitioner”: http://www.gikids.org/files/documents/resources/IBD-TransitionTeenIBD.pdf	
*For providers*	
Healthcare provider checklist for transitioning a patient with IBD from pediatric to adult care [20]	
“Transitioning a patient with IBD from pediatric to adult care”: http://www.gikids.org/files/documents/resources/Checklist_ONLYHealthcareProdiver_TransitionfromPedtoAdult.pdf	
**•** TRxANSITION scale and STARx transition readiness questionnaire [10]	
***Health passports, self-management tools, and symptom trackers***	
**•** Good 2 Go Transition Program – MyHealth Passport: https://www.sickkids.ca/myhealthpassport/	
***Resources for adolescents and parents***	
**•** Crohn’s and Colitis Foundation Campus Connection: https://www.crohnscolitisfoundation.org/campus-connection	
**•** ImproveCareNow: https://improvecarenow.org	
**•** Just Like Me: https://www.crohnscolitisfoundation.org/justlikeme **•** Doc4me app: http://www.doc4me-app.com/	
***Transition advocacy and support for patients, parents, and providers***	
**•** “Got Transition/Center for Health Care Transition”: http://gottransition.org/	
**•** The Society of Adolescent Health and Medicine: http://www.adolescenthealth.org/Home.aspx	

Regardless of the checklist utilized, the consistent message from these tools is a) starting the assessment as early as 11-12 years of age, b) the knowledge needs to be assessed in an age-appropriate manner, and c) should be a process that involves repeated assessment over time, leading up to the transfer of care to the adult gastroenterologist.

## Self-efficacy Skills

Development of self-efficacy skill, or the belief in one’s ability to execute and decide on new challenges presented in their medical care, have been shown to be a key component for a successful transition of care for adolescents and young adults with chronic illnesses [21]. The IBD-yourself tool was developed by Ziljlstra et. al to allow for assessment of self-efficacy skills in adolescent IBD patients (Same as above reference). In a follow up study by Yershalmy-Feler et al utilizing the IBD-yourself tool, they demonstrated a significant increase in self-efficacy skills for adolescent IBD patients from 1.41 +- 0.21 to 1.85 +- 0.3 (p < 0.0001) [22]. In addition to the IBD-yourself, validation data has been published on the self-efficacy tool entitled the IBD Self-Efficacy Scale for Adolescents and Young Adults (IBDSES-A) [23].

## Disease Knowledge Assessment

Assessment of disease knowledge can benefit disease management by identifying or correcting patient misconceptions about IBD [24]. Eaden et. al developed the Crohn’s and Colitis Knowledge (CCKNOW) Score which is a tool covering general concepts in IBD and can be used in clinical practice to assess disease knowledge [25]. One of the limitations of the CCKNOW score is the development and validation was not done in a pediatric specific population. In contrast, the recently developed IBD-Knowledge Inventory Device (IBD-KID) tool was developed and validated in a pediatric specific population and can be used to assess disease related knowledge [26]. This tool was recently revised to IBD-KID2, which is a fifteen-item knowledge assessment aimed to provide a more simplified tool which could be used in children with IBD down to 8 years of age [24]. A separate study found that more patients preferred IBD-KID2 to CCKNOW and was also easier to complete suggesting there may be lower burden to the participants leading to higher completion rates [27].

## Transition Readiness

As the young adult approaches the transfer of care to the adult gastroenterologist, there is a need to assess overall transition readiness as they prepare for this change. A commonly used tool for this is the Transition Readiness Assessment Questionnaire (TRAQ) [28, 29]. The TRAQ focuses on patients and their knowledge in realms including medication management, appointments, health issue tracking, activities of daily life, and communication with the healthcare team [28, 29]. The questionnaire has twenty items that can be administered in the clinical setting for patients 12 years and older and includes the following sections: 1) Managing Medications; 2) Appointment Keeping; 3) Tracking Health Issues; 4) Talking with Providers; and 5) Managing Daily Medications. The score ranges from 0-100, with a higher score denoting higher transition readiness. Recent studies have utilized the TRAQ higher scores, hence higher transition readiness, when adolescents and young adults attend a structured transition clinic as compared to when they do not attend such a clinic [30, 31]. Another tool used in clinical practice is the UNC Transition Score (TR_x_ANSITION Scale™), which is a 33-item, ten domain questionnaire that has been validated for use in children 12 years and older [32]. Finally, “Got Transition®” utilizes questions for patients and parents/caregivers in areas including health management and skills that can be used in health care. (Reference: https://www.gottransition.org/) These tools can be incorporated within certain electronic medical record (EMR) systems depending on the institution.

## Transfer Resources

One IBD-specific transfer resource available to assist both the pediatric gastroenterology team and the patient to find an adult gastroenterologist with specific expertise in the care of IBD patients is the “Doc4Me” smartphone application developed by Jeannie Huang, MD and promoted by NASPGHAN (https://www.doc4me-app.com/). This application allows patients to access a curated nationwide database of adult gastroenterologists who specialize in the care of IBD patients by their current geographic location, along with other helpful resources such as provider reviews and a built-in transition checklist.

## Summary

### Young adult patients

There are key issues that we need to ensure our young adult patients living with IBD needs to know: administrative issues (accommodations in college, internships, and work); insurance mandates (know your coverage; understand the difference between insurance versus PBM; what does the prior authorization process entail for your therapies); health management (how to schedule your visits and tests; where do you get your medications and how should you use them; how and when to contact your healthcare team; emergency contacts and supports) and health maintenance (understand medications and interactions; understand and incorporate self-protection in the time of viral seasons and pandemics; sexual health and planning). Young adult patients also need to know they are not alone. There are numerous peer support groups including the Crohn’s and Colitis Foundation (https://www.crohnscolitisfoundation.org/campus-connection/managing-your-care/beyond-college). Crohn’s and Colitis Young Adult Network (https://www.ccyanetwork.org), and Improve Care Now (https://www.improvecarenow.org/patients-parents).

### Clinicians

It is important to recognize that transition is a gradual process – “continuum of care” that allows the adolescent and young adult patient to eventually achieve self-efficacy and autonomy in their medical care, and overall health management. Furthermore, it is most successful when all stakeholders – the patient, family, and healthcare team – work together. The most effective therapeutic alliance is the one where there is shared decision making, open communication and a safe space for the patient. It is important to provide appropriate and effective tools and resources within the healthcare system itself as well as on a global level. Transition and transfer of medical care coincides with other life transitions. Therefore, all aspects of the patient (and family) life need to be considered.

In this process, empower your emerging adult patients; acknowledge the challenges they encounter as they develop their own independent identity as a young adult living with a chronic illness. By doing so, the care team will provide young adult patients living with IBD the ability to be effective in their lifelong care.

## Abbreviations

AYA: Adolescent/young adult
IBD: Inflammatory bowel diseases
UC: Ulcerative colitis
